# Does mild COPD affect prognosis in the elderly?

**DOI:** 10.1186/1471-2466-10-35

**Published:** 2010-06-07

**Authors:** Claudio Pedone, Simone Scarlata, Claudio Sorino, Francesco Forastiere, Vincenzo Bellia, Raffaele Antonelli Incalzi

**Affiliations:** 1 Centro per la Salute dell'Anziano, Area di Geriatria. Università Campus Biomedico, Roma, Italy; 2 Fondazione Alberto Sordi, Roma, Italy; 3 Dipartimento di Medicina, Pneumologia, Fisiologia e Nutrizione Umana. Università di Palermo, Palermo, Italy; 4 Dipartimento di Epidemiologia, ASL RM-E. Roma, Italy; 5 Fondazione San Raffaele - Cittadella della Carità. Taranto, Italy

## Abstract

**Background:**

Chronic obstructive pulmonary disease (COPD) affects independence and survival in the general population, but it is unknown to which extent this conclusion applies to elderly people with mild disease. The aim of this study was to verify whether mild COPD, defined according to different classification systems (ATS/ERS, BTS, GOLD) impacts independence and survival in elderly (aged 65 to 74 years) or very elderly (aged 75 years or older) patients.

**Methods:**

We used data coming from the Respiratory Health in the Elderly (Salute Respiratoria nell'Anziano, SaRA) study and compared the differences between the classification systems with regards to personal capabilities and 5-years survival, focusing on the mild stage of COPD.

**Results:**

We analyzed data from 1,159 patients (49% women) with a mean age of 73.2 years (SD: 6.1). One third of participants were 75 years or older. Mild COPD, whichever was its definition, was not associated with worse personal capabilities or increased mortality after adjustment for potential confounders in both age groups.

**Conclusions:**

Mild COPD may not affect survival or personal independence of patients over 65 years of age if the reference group consists of patients with a comparable burden of non respiratory diseases. Comorbidity and age itself likely are main determinants of both outcomes.

## Background

Chronic obstructive lung disease has as key feature the reduction of forced expiratory volume in 1 second (FEV1) relative to the vital capacity (VC) or to the forced vital capacity (FVC), and various cut-off of the FEV1/FVC ratio have been proposed to diagnose this disease. According to the British Thoracic Society and the UK National Institute for Clinical Excellence (NICE) a diagnosis of COPD requires both FEV1/FVC < 0.7 and FEV1 < 80% predicted [1], while the Global initiative for Obstructive Lung Disease (GOLD) guidelines proposes a FEV1/FVC *<*0.7 as diagnostic standard, classifying as GOLD class II those also having a FEV1 < 80% predicted. The 2004 European Respiratory Society (ERS)/American Thoracic Society (ATS) guidelines also use a fixed cut-off of 0.7 for the FEV1/VC ratio [2], but a statement on interpretation of spirometry issued in 2005 strongly suggests to use the lower limit of normal (LLN) to make a diagnosis of bronchial obstruction [3]. In the same guidelines, severity classification is based on FEV1% predicted, with a cut-off for the first (mild) class set at 70%.

Further classification issues arise in elderly patients: in this population the use of the 0.7 fixed cut-off to diagnose bronchial obstruction has been criticized because it overestimates disease prevalence compared to the LLN [4], and a lower cut-off (0.65) has been proposed [5]. Data from the Cardiovascular Health Study, however, show that elderly people with FEV1/FVC < 0.7 but *>*LLN have an increased risk of death compared to their counterpart with FEV1/FVC ≥ 0.7, lending support to the use of a fixed cut-off set at 0.7 in this population [6].

Data on the prognostic significance of the different classification methods are scant, especially with respect to the "mild" category. The interest in the "mild" category comes also from the fact that two out of the three most commonly used guidelines include in this classification patients with a normal FEV1. Since the FEV1 is the physiologic parameter of interest in COPD and it has been shown to have important prognostic implications [7], it is reasonable to question what is the prognosis of people classified as having mild obstruction according to the ATS/ERS or GOLD classification (i.e. with normal FEV1) compared to those classified as having mild obstruction according to the BTS classification (i.e. with reduced FEV1). On this basis, we analyzed data coming from a large collaborative study on respiratory health in elderly people (SaRA Study) to verify the association between mild COPD, defined according to different algorithms, and mortality and functional status in a sample of elderly (age 65 up to 75 years) and very elderly (age ≥ 75 years) patients.

## Methods

### Data source

Between January 1996 and July 1999 a total of 1970 out-patients were recruited from 24 departments of geriatrics or respiratory medicine within the context of the SA.R.A. (Salute Respiratoria nell'Anziano - Respiratory Health in the Elderly) study. Details on the SA.R.A. project are available elsewhere [8]. This is a multi-centre Italian project, investigating various aspects of chronic airway diseases in the elderly population (age ≥65 years) attending pulmonary or geriatric outpatient clinics. Researchers had an extensive training in both respiratory function study of the elderly and multidimensional geriatric assessment. Enrolment was on a consecutive basis up to the achievement of a target number of about 200 COPD and 200 asthmatic patients. The study also enrolled as a control group outpatients aged 65 years and over attending the geriatric clinics for non respiratory conditions, the most common of which were: hypertension (27.3%), arthritis (27%), diabetes mellitus (12.7%), coronary artery disease (11.4%), and cerebrovascular diseases (7%). Data from individual centres were collected by a coordinating centre at the Cattedra di Malattie dell'Apparato Respiratorio of the University of Palermo, which was also responsible for the quality control, the retrieval and the final processing of data. The study design was approved by the Ethical Committees of University of Palermo and of the participating institutions. Patients gave their written consent to participate in the study.

### Pulmonary function tests

All the centres were provided with an identical fully computerized water-sealed Stead-Wells spirometer (Baires System; Biomedin; Padua, Italy) matching the standards of the American Thoracic Society recommendations for diagnostic spirometry. Baseline and post-bronchodilator spirometry were performed according to the guidelines of the American Thoracic Society [9]. All the centers achieved a high quality performance in spirometry. Spirometric flow-volume curves were considered acceptable if they had Extrapolated Volume (VEXT) *<*5% of the FVC or *<*0.150 L, a FET *>*6 sec and a plateau of 1 second, or a FET *>*10 sec. According to recommendations by ATS, we did not exclude curves which did not satisfy the repeatability criteria to avoid the exclusion of data in which an abnormal lung function causes a greater coefficient of variation than in normal subjects. Overall, 1,759 spirometries were available for the analysis. Reasons for not performing the exam included refusal, physical impairment, and inability to understand the instructions.

### Sample selection and follow-up

We excluded participants with spirometries that did not meet acceptability criteria (N = 382). We also excluded participants with missing anthropometric values (N = 8). Vital status information as of January 30, 2002 was ascertained by contacting the registry office of the last municipality of residence, and the data was obtained for 1159/1369 participants (85%). Follow-up time was calculated from the date of recruitment (first visit) until the date of death, January 30, 2002, or at 60 months of follow-up, whichever came first.

### Analytic approach

We used three definitions of mild bronchial obstruction based on current guidelines:

1. FEV1/FVC *<*LLN; FEV1 *>*70% predicted (ATS/ERS)

2. FEV1/FVC *<*0.7; FEV1 between 50% and 80% predicted (BTS/NICE)

3. FEV1/FVC *<*0.7; FEV1 ≥ 80% predicted (GOLD)

Participants could of course fall into more than one definition, and were counted into each.

Thus, the study population consisted of patients meeting at least one of the three definitions of bronchial obstruction, while the control population included ambulatory patients without respiratory problems. Accordingly, we evaluated the prognostic significance of mild COPD against a control group representative of the general elderly population. We used descriptive statistics to report the demographic and clinical characteristics of the three groups of patients with mild COPD. Performance capacity was evaluated using the distance walked in 6 minutes (6MWD), expressed as % of predicted according to the equation proposed by Enright [10]. Cognition and mood status were explored using the Mini-mental State Examination (MMSE) and the Geriatric Depres-sione Scale (GDS), respectively, while as a reference indicator of prognosis we used a validated score based on body mass index, severity of bronchial obstruction, dyspnea, and exercise capacity (BODE index) [7].

We calculated mortality rated normalized to 100 person/year (100 PY) along with 95% confidence intervals (95% CI). For each definition, we calculated the mortality rate ratio using as control group participants who were non-obstructed according to the relevant classification. We also obtained an estimate of the hazard ratio for mortality adjusted for age, gender, smoking status and FVC using a Poisson model. We used this parametric approach instead of the more commonly used Cox model because preliminary analyses showed that the proportional hazard assumption was not tenable.

In order to assess the impact of mild COPD on health status, we compared the 6MWD, Barthel index, MMSE, and GDS of control subjects and COPD patients alternatively grouped according to each of the three classifications. We used ANOVA for data having normal distribution and homogenous variance, the Kruskal-Wallis test otherwise.

All analyses were performed separately in participants aged *<*75 and ≥ 75 years using SAS 9.0 for Windows (SAS Inc, Cary NC) and Stata 10.0 for Windows (StataCorp, College Station TX).

## Results

### Characteristics of the sample

The final sample size was 1,159, women were 48.7%, the mean age was 73.2 (SD: 6.1), and 429 participants (37.1%) were 75 years or older. The overall prevalence of bronchial obstruction was 40.0%, 28.5%, and 43.6% according to the ATS/ERS, BTS and GOLD definitions, respectively. Table 1 shows the prevalence of bronchial obstruction according to the different definitions. Among participants aged 65 to 75 years, mild obstruction was present in 18.8%, 18.5%, and 10.8% using the ATS/ERS, BTS, and GOLD classification, respectively. The prevalence of mild obstruction among older participants was similar using the ATS/ERS and BTS classifications, while using the GOLD definition the figure was doubled (22.4%) compared to the one observed in the group aged 65-75 years.

**Table 1:** Prevalence of bronchial obstruction according to different classification systems, by age

	Age 65 - 74 years N (%)	Age ≥ 75 years N (%)
ATS/ERS		
No obstruction	451 (61.8)	245 (57.1)
Mild obstruction	137 (18.8)	93 (21.7)
Moderate/severe obstruction	142 (19.4)	91 (21.1)

BTS		
No obstruction	527 (72.2)	302 (70.4)
Mild obstruction	135 (18.5)	96 (22.4)
Moderate/severe obstruction	68 (9.3)	31 (7.2)

GOLD		
No obstruction	448 (61.4)	206 (48.0)
Mild obstruction	79 (10.8)	96 (22.4)
Moderate/severe obstruction	203 (27.8)	127 (29.6)

There were few differences in the prevalence of major diseases across COPD severity regardless of the classification systems; however in patients under 75 years the BTS-rated mild COPD was associated with a greater prevalence of ischemic heart disease with regard to non obstructed patients, while the opposite was true for the GOLD-rated mild COPD. Also indexes of personal capabilities, cognitive and affective status of controls and cases alternatively classified according to each of the three classifications showed no clinically significant differences between people without obstruction and with mild obstruction (tables 2 and 3).

**Table 2:** Clinical and functional characteristics, by age and severity of obstruction.

	ATS/ERS stage		
	No Obstruction	Mild obstruction	Moderate/Severe obstruction
	
Males (%)	41.2	59.8	68.3
Ischemic heart disease (%)	13.0	13.2	19.9
Cerebro-vascular disease (%)	4.5	3.7	6.4
Diabetes (%)	11.7	9.6	12.1
Cancer (%)	4.0	3.7	4.3
6MWD % predicted (mean [SD])	81.0 (26.3)	78.2 (24.0)	68.6 (24.2)
GDS score (mean [SD])	3.5 (3.3)	2.8 (3.1)^1^	3.7 (3.3)
MMSE score (mean [SD])	27.7 (3.0)	27.4 (2.8)	26.7 (4.2)
Barthel Index (mean [SD])	95.0 (7.9)	95.8 (5.0)^1^	92.8 (7.6)
	**BTS stage**		
	**No Obstruction**	**Mild obstruction**	**Moderate/Severe obstruction**
	
Males (%)	43.3	63.0	76.5
Ischemic heart disease (%)	12.4	22.0	14.7
Cerebro-vascular disease (%)	4.4	5.3	11.8
Diabetes (%)	10.9	12.1	13.2
Cancer (%)	3.8	6.1	1.5
6MWD % predicted (mean [SD])	80.6 (26.2)	76.3 (21.7)	62.8 (25.3)
GDS score (mean [SD])	3.5 (3.3)	3.2 (3.2)	3.5 (3.0)
MMSE score (mean [SD])	27.7 (2.9)	26.9 (3.9)^1^	26.8 (4.2)
Barthel Index (mean [SD])	96 (7.7)	95.2 (4.8)	91.5 (8.5)

	**GOLD stage**		
	**No Obstruction**	**Mild obstruction**	**Moderate/Severe obstruction**
	
Males (%)	40.4	59.5	67.5
Ischemic heart disease (%)	13.5	6.4	19.5
Cerebro-vascular disease (%)	4.5	3.8	5.5
Diabetes (%)	11.7	6.4	12.5
Cancer (%)	4.0	2.6	4.5
6MWD % predicted (mean [SD])	81.3 (26.2)	77.5 (26.0)	71.1 (23.9)
GDS score (mean [SD])	3.6 (3.3)	2.9 (3.3)^1^	3.3 (3.2)
MMSE score (mean [SD])	27.7 (3.0)	27.7 (2.6)	26.8 (4.0)
Barthel Index (mean [SD])	94.9 (8.0)	95.8 (5.7)^1^	94.0 (6.5)

Age *<*75 years.

^1^P < 0.05.

**Table 3:** Clinical and functional characteristics, by age and severity of obstruction.

	ATS/ERS stage		
	No Obstruction	Mild obstruction	Moderate/Severe obstruction
	
Males (%)	47.3	58.1	65.9
Ischemic heart disease (%)^1^	21.6	23.1	25.6
Cerebro-vascular disease (%)	11.0	5.6	4.4
Diabetes (%)	15.9	15.4	16.7
Cancer (%)	8.2	8.8	4.4
6MWD % predicted (mean [SD])	76.8 (30.5)	78.1 (24.7)	66.2 (24.9)
GDS score (mean [SD])	4.2 (3.3)	3.6 (3.4)	4.5 (3.5)
MMSE score (mean [SD])	26.5 (3.7)	26.4 (4.2)	25.3 (4.8)
Barthel Index (mean [SD])	92.8 (9.6)	92.6 (12.4)	91.0 (10.9)
	**BTS stage**		
	**No Obstruction**	**Mild obstruction**	**Moderate/Severe obstruction**
	
Males (%)	47.3	66.7	74.2
Ischemic heart disease (%)^1 ^21.9	24.5	25.8	
Cerebro-vascular disease (%)	10.0	5.3	3.2
Diabetes (%)	16.6	16.0	9.7
Cancer (%)	8.3	6.4	3.2
6MWD % predicted (mean [SD])	78.9 (29.0)	69.9 (24.0)^1^	57.8 (26.8)
GDS score (mean [SD])	4.0 (3.3)	4.2 (3.5)	5.2 (4.1)
MMSE score (mean [SD])	26.5 (3.8)	25.6 (4.6)^1^	25.6 (4.5)
Barthel Index (mean [SD])	92.9 (10.3)	91.7 (9.9)	89.7 (14.1)

	**GOLD stage**		
	**No Obstruction**	**Mild obstruction**	**Moderate/Severe obstruction**
	
Males (%)	42.7	57.3	68.5
Ischemic heart disease (%)^1^	22.3	21.0	24.8
Cerebro-vascular disease (%)	11.2	7.4	4.8
Diabetes (%)	16.5	16.8	14.4
Cancer (%)	7.8	9.5	5.6
6MWD % predicted (mean [SD])	81.3 (29.4)	75.2 (28.0)	66.8 (25.1)
GDS score (mean [SD])	4.1 (3.6)	3.6 (3.3)	4.4 (3.7)
MMSE score (mean [SD])	26.7 (3.6)	26.1 (4.3)	25.6 (4.5)
Barthel Index (mean [SD])	93.3 (9.3)	92.1 (12.2)	91.2 (11.0)

Age ≥ 75 years.

^1^P < 0.05.

### Mortality data in younger participants

**Figure 1:**
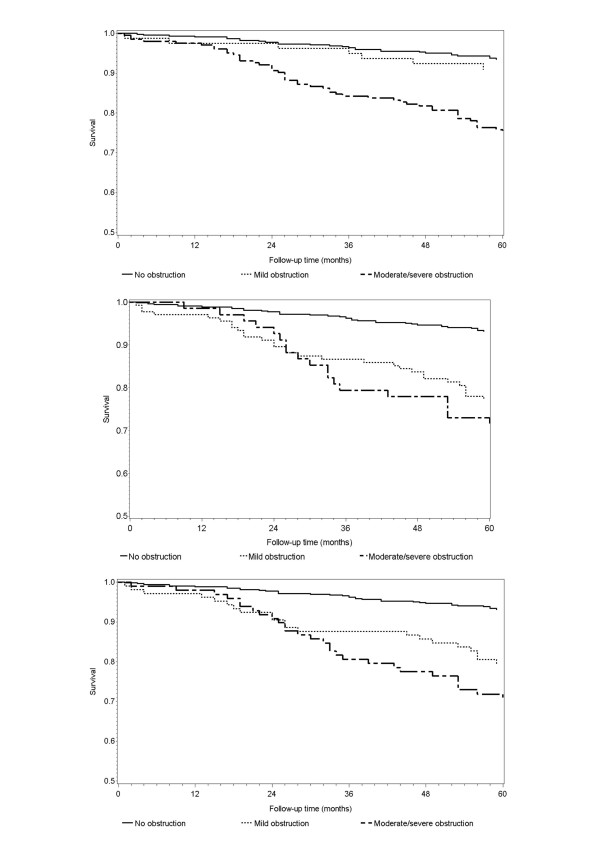
**Survival by COPD severity in patients aged 65-74 years (unadjusted Kaplan-Meier curves)**. Upper panel: ATS/ERS classification; middle panel: BTS classification; lower panel: GOLD classification. Log-rank P < 0.001.

In this group, the overall mortality was 2.5/100 person/year (95% CI: 1.98 - 3.08), with substantial difference between men (MR: 3.6/100 PY, 95% CI: 2.72 - 4.62) and women (MR: 1.4/100 PY, 95% CI: 0.94 - 1.14). The most common causes of death were tumors and cardio-vascular diseases; pulmonary diseases accounted for 6% of cases. Moderate/severe obstruction was associated with an about four-fold mortality regardless of the classification system (table 4 and figure 1). Mortality associated with mild obstruction, however, showed differences according to different definitions: mortality was increased (MRR: 3.62, 95% CI: 2.14 - 6.07) using the BTS and, less markedly (MRR 1.97; 95% CI: 1.01 - 3.70), the ATS classification, while we found no increase in mortality associated with mild obstruction defined according to the GOLD criteria (MRR: 1.42, 95% CI: 0.52 - 3.34). After adjustment for age, gender, smoking exposure and FVC, the MRR for mortality associated with BTS-rated mild obstruction was no more statistically significant, while those associated with more severe obstruction were reduced, but still statistically significant.

**Table 4:** Crude and adjusted mortality rate ratios (MRR) associated with severity of obstruction according to different classification systems, by age.

	Crude MRR		Adjusted^1 ^MRR	
	
	**Age < 75 years**	Age ≥ 75 years	Age < 75 years	Age ≥ 75 years
ATS				
Mild obstruction	1.97 (1.01-3.70)	0.75 (0.43-1.25)	1.70 (0.92-3.18)	0.80 (0.48-1.33)
Moderate/severe obstruction	4.62 (2.78-7.76)	1.71 (1.11-2.58)	2.64 (1.49-4.68)	1.31 (0.83-2.10)

BTS				
Mild obstruction	3.62 (2.14-6.07)	2.52 (1.50-4.25)	1.28 (0.80-1.98)	1.05 (0.66-1.68)
Moderate/severe obstruction	4.68 (2.53-8.41)	2.83 (1.43-5.60)	3.03 (1.73-5.06)	2.01 (1.07-3.98)

GOLD				
Mild obstruction	1.42 (0.52-3.34)	0.97 (0.57-1.50)	1.21 (0.51-2.87)	0.98 (0.60-1.61)
Moderate/severe obstruction	4.22 (2.60-6.98)	1.64 (1.09-2.47)	2.67 (1.60-4.48)	1.19 (0.77-1.85)

^1^Adjusted for age, gender, smoking exposure, and FVC.

### Mortality data in older participants

**Figure 2:**
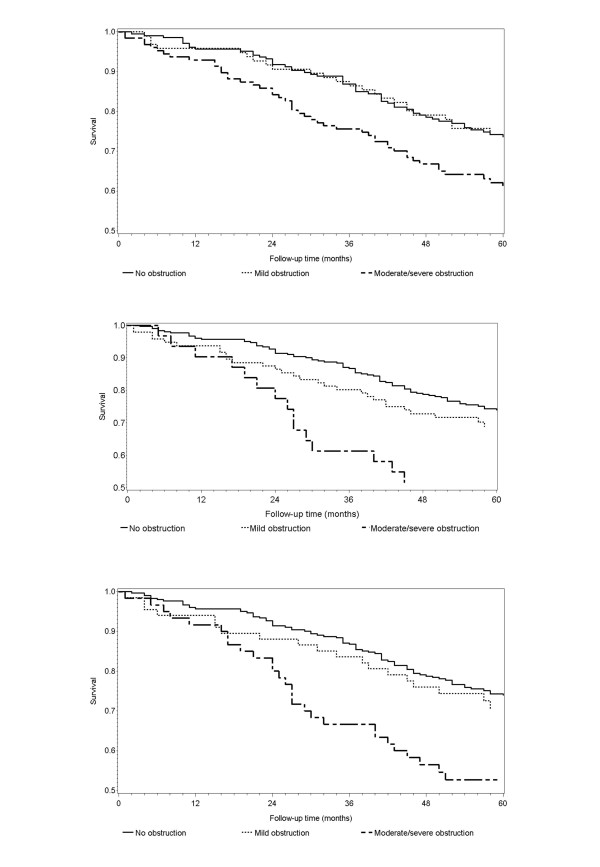
**Mortality by COPD severity in patients aged ≥ 75 years (unadjusted Kaplan-Meier curves)**. Upper panel: ATS/ERS classification; middle panel: BTS classification; lower panel: GOLD classification. Log-rank P = 0.003.

As expected, the overall mortality of older participants was higher, 6.9/100 person/year (95% CI: 5.68 - 8.28). Once again, mortality was higher in men (MR: 8.7/100 PY, 95% CI: 6.95 - 10.88) compared to women (MR: 5.0/100 PY, 95% CI: 3.67 - 6.77). The most common cause of death were cardio-vascular diseases (31.7%) and tumors (25.2%); pulmonary diseases accounted for 13.0% of deaths. Only mild obstruction defined according to the BTS criteria was associated with increased mortality (table 4 and figure 2) in this group. More severe obstruction was associated with mortality, but the risk was increased less compared to patients aged 65-75 years; severe obstruction defined according to the BTS criteria was associated with the greatest increase in risk (MRR: 2.83, 95% CI: 1.43 - 5.60). After correction for potential confounders, only severe COPD as defined by the BTS criteria was still associated with mortality (MRR: 2.01; 95% CI: 1.07 - 3.98). Since in older patients mortality associated with COPD might be reduced because of competing risk, we evaluated whether another severity indicator (the BODE index) was associated with mortality in this age group and found an adjusted HR of 1.26 (95% CI: 1.14 - 1.41) for each one-point increase in the BODE score.

## Discussion

Our results confirm that the estimated prevalence of COPD depends on the definition used, but "mild" COPD, regardless of the definition used, impacts neither prognosis nor personal capabilities in an elderly population.

Clinical diagnosis of COPD should not rely on spirometry alone, but a positive spirometry is required by all guidelines to make a definitive diagnosis. For this reason, and also for practicality, most epidemiological studies use spirometry to identify people with COPD, making the issue of spirometric definition of bronchial obstruction an important one. As expected, the GOLD guidelines yielded the highest estimated prevalence of COPD and the BTS the lowest, a result that is also in line with other studies [11,12]. Diagnostic algorithms using a fixed cut-off to identify bronchial obstruction are expected to provide higher prevalence estimates in the elderly compared to algorithms using the LLN; in our data, however, this was true for the GOLD but not for the BTS classification. The likely explanation for this finding is that the BTS classification system also requires a reduction of the FEV1%. On the other hand, it cannot be excluded that the prevalence of COPD in elderly people is actually higher, as shown by different authors [13,14]. It should be noted, however, that most of these data have been obtained in studies defining COPD using a fixed cut-off, and no data are available on the age-specific prevalence of COPD defined using the LLN.

The use of the LLN is only partial solution to these problems. The LLN is population-specific, and ideally should be calculated on a subset of healthy, non-smoking persons of the population under study, which is obviously hardly feasible in most studies and in the clinical setting. Using LLN derived from external studies is easier, but they can have a wide variability [15] and their use also carries a significant risk of misclassification [16,17].

In participants aged 65-75 years, we have found that, after adjustment for potential confounders, mild COPD is not associated with increased mortality, but this finding should be interpreted also taking into account the relatively small sample size of our study. Mannino et al. have shown in a population aged up to 66 years that the adjusted hazard ratio for mortality in GOLD stage 1 COPD was 1.4 (95% CI: 1.1 - 1.6), which is close to the 1.2 we found in our sample [18]. In another large screening study, among middle-aged men the hazard ratio for mortality associated with GOLD 1 ranged from 0.8 to 1.3, depending on smoking exposure [19]. There are scant data on the risk of mortality in patients with mild COPD as defined by the ATS/ERS criteria; it has been shown, however, that people aged 65 years and older with FEV1/FVC *<*LLN and FEV1 ≤ 80% of predicted have an adjusted mortality rate ratio of 1.4 [6].

Interestingly, in the group aged 75 years and older, we found no association between moderate/severe COPD (ATS/ERS and GOLD classification) and mortality. This finding likely reflects the preeminent prognostic role of non respiratory factors in older people, even with severely reduced FEV1. Among these factors are age itself, comorbidity and performance capacity [20]. Indeed, we found that the BODE Index was associated with mortality in this age group, indicating that a multidimensional index is effective also for the very old. Further supporting the primary role of multidimensional assessment in contrast to the monodimensional FEV1-based assessment is the finding that people with respiratory symptoms, but without spirometric abnormalities, have a worse prognosis [18,19]. Furthermore, symptomatic, but not asymptomatic, patients with GOLD stage 1 COPD have faster FEV1 decline, increased respiratory care utilization, and worse quality of live [21]. Even in GOLD stages 2-3, symptoms seem to have important prognostic implication over and above spirometric abnormalities [19]. Thus, the global burden of disease rather than bronchial obstruction seems relevant to predict prognosis of elderly COPD patients. At any rate, it should be noted that the relationship between FEV1 reduction and mortality is a complex one, as shown by recent randomized trials showing that improvement in FEV1 does not translate in increased survival [22,23].

In this sample, personal capabilities, affective and cognitive status of mild COPD patients did not differ from those of control subjects with comparable non respiratory diseases, indicating that comorbidity, more than mild COPD, likely accounts for impaired health status. A study by Coultas et al. [24], however, have found impaired personal capabilities even in COPD subjects unaware of having the disease, which is consistent with an direct association between mild COPD and health status. Differences in mean age between ours (73.2 years) and Coultas' patients (65.7 years) might explain this discrepancy. Furthermore, our controls were not healthy subjects, but patients with non respiratory conditions. This likely enabled us to disclose the relationship between comorbidity and personal capabilities.

Finally, physical performance measured by the 6MWD was not different between participants with mild COPD defined by the three alternative classifications, although those diagnosed according to the BTS criteria have a lower FEV1. This is confirmatory of previous studies showing that the FEV1 is not the best predictor of performance capacity in COPD [25,26] and that patients with mild disease defined using the GOLD criteria have a performance similar to that of a control group free from pulmonary diseases [27].

This study is limited by its relatively low power and by the fact that its 5 years follow-up time may be too short to detect important differences in mortality in people having mild COPD. It should be noted, however, that the mean age of our older participants -- which was the group of major interest -- was 80 years, and in a group with such an age five years should suffice to provide information on mortality. It should be also noted that the selection of patients from health care facilities might limit the representativeness of the sample. Furthermore, we had no generic index of health-related quality of life recorded in the whole population and we cannot exclude that mild COPD affects this outcome. On the other hand, the availability of a control group with comparable age and comorbidity improves the reliability of our estimates of the prognostic properties of mild COPD. Finally, a consistent proportion of participants could not perform an acceptable spirometry. This information is not new, and we have already reported [8] that elderly patients unable perform an acceptable spirometry have worse physical and cognitive performance, and lower education and therefore have an inherently poorer prognosis. In theory, obtaining acceptable spirometric information also in these patients (e.g. using the FEV6 instead of the FVC) might show a poorer prognosis even in those with mild obstruction.

## Conclusions

Our data indicate that mild COPD may not affect survival and personal capabilities of patients over 65 years, regardless of the criteria used to diagnose it. In this group of patients, comorbidity and age itself likely are the major determinants of these outcomes.

## Competing interests

The authors declare that they have no competing interests.

## Authors' contributions

CP and SS had the original idea for the paper. CP and FF planned the statistical analyses. CP, CS and RAI drafted the manuscript. All the authors revised the paper for important intellectual content.

## Pre-publication history

The pre-publication history for this paper can be accessed here:

http://www.biomedcentral.com/1471-2466/10/35/prepub

## References

[B1] Diagnosing COPD Thorax 2004 59 90001 i27 38 http://thorax.bmj.com/content/59/suppl_1

[B2] Celli BR MacNee W Standards for the diagnosis and treatment of patients with COPD: a summary of the ATS/ERS position paper The European Respiratory Journal: Official Journal of the European Society for Clinical Respiratory Physiology 2004 23 6 932 46 PMID: 15219010. 15219010 10.1183/09031936.04.00014304

[B3] Pellegrino R Viegi G Brusasco V Crapo RO Burgos F Casaburi R Coates A van der Grinten CPM Gustafsson P Hankinson J Jensen R Johnson DC MacIntyre N McKay R Miller MR Navajas D Pedersen OF Wanger J Interpretative strategies for lung function tests The European Respiratory Journal: Official Journal of the European Society for Clinical Respiratory Physiology 2005 26 5 948 68 [PMID: 16264058]. 16264058 10.1183/09031936.05.00035205

[B4] Roberts SD Farber MO Knox KS Phillips GS Bhatt NY Mastronarde JG Wood KL FEV1/FVC ratio of 70% misclassifies patients with obstruction at the extremes of age Chest 2006 130 200 6 [PMID: 16840402]. 10.1378/chest.130.1.200 16840402

[B5] Medbö A Melbye H Lung function testing in the elderly-Can we still use FEV1/FVC <70% as a criterion of COPD? Respiratory Medicine 2007 101 6 1097 1105 http://www.sciencedirect.com/science/article/B6WWS-4MVDVFR-1/2/96e542a12adc0f25465ff7aaa0cf76aa 10.1016/j.rmed.2006.11.019 17239575

[B6] Mannino DM Buist AS Vollmer WM Chronic obstructive pulmonary disease in the older adult: what defines abnormal lung function? Thorax 2007 62 3 237 241 [PMID: 17090573]. 10.1136/thx.2006.068379 17090573 PMC2117148

[B7] Celli BR Cote CG Marin JM Casanova C de Oca MM Mendez RA Plata VP Cabral HJ The body-mass index, airflow obstruction, dyspnea, and exercise capacity index in chronic obstructive pulmonary disease The New England Journal of Medicine 2004 350 10 1005 1012 [PMID: 14999112]. 10.1056/NEJMoa021322 14999112

[B8] Bellia V Pistelli R Catalano F Antonelli-Incalzi R Grassi V Melillo G Olivieri D Rengo F Quality control of spirom-etry in the elderly. The SA.R.A. study. SAlute Respiration nell'Anziano = Respiratory Health in the Elderly American Journal of Respiratory and Critical Care Medicine 2000 161 4Pt1 1094 100 [PMID: 10764296]. 10764296 10.1164/ajrccm.161.4.9810093

[B9] Standardization of Spirometry, 1994 Update. American Thoracic Society American Journal of Respiratory and Critical Care Medicine 1995 152 3 1107 36 [PMID: 7663792] 7663792 10.1164/ajrccm.152.3.7663792

[B10] Enright PL Sherrill DL Reference equations for the six-minute walk in healthy adults American Journal of Respiratory and Critical Care Medicine 1998 158 5 Pt 1 1384 1387 [PMID: 9817683]. 9817683 10.1164/ajrccm.158.5.9710086

[B11] Celli BR Halbert RJ Isonaka S Schau B Population impact of different definitions of airway obstruction The European Respiratory Journal: Official Journal of the European Society for Clinical Respiratory Physiology 2003 22 2 268 73 [PMID: 12952259]. 12952259 10.1183/09031936.03.00075102

[B12] Lindberg A Jonsson A Rönmark E Lundgren R Larsson L Lundbäck B Prevalence of chronic obstructive pulmonary disease according to BTS, ERS, GOLD and ATS criteria in relation to doctor's diagnosis, symptoms, age, gender, and smoking habits Respiration; International Review of Thoracic Diseases 2005 72 5 471 9 [PMID: 16210885]. 16210885 10.1159/000087670

[B13] Hansen JG Pedersen L Overvad K Omland Ø Jensen HK Sørensen HT The Prevalence of chronic obstructive pulmonary disease among Danes aged 45-84 years: population-based study Copd 2008 5 6 347 52 [PMID: 19353348]. 10.1080/15412550802522635 19353348

[B14] Lindberg A Bjerg A Bjerg-Bäcklund A Rönmark E Larsson L Lundbäck B Prevalence and underdiagnosis of COPD by disease severity and the attributable fraction of smoking Report from the Obstructive Lung Disease in Northern Swe-den Studies Respiratory Medicine 2006 100 2 264 272 [PMID: 15975774]. 10.1016/j.rmed.2005.04.029 15975774

[B15] Swanney MP Ruppel G Enright PL Pedersen OF Crapo RO Miller MR Jensen RL Falaschetti E Schouten JP Hankinson JL Stocks J Quanjer PH Using the lower limit of normal for the FEV1/FVC ratio reduces the misclassification of airway obstruction Thorax 2008 63 12 1046 1051 [PMID: 18786983]. 10.1136/thx.2008.098483 18786983

[B16] Kuster SP Kuster D Schindler C Rochat MK Braun J Held L Brändli O Reference equations for lung function screening of healthy never-smoking adults aged 18-80 years The European Respiratory Journal: Official Journal of the European Society for Clinical Respiratory Physiology 2008 31 4 860 868 [PMID: 18057057]. 18057057 10.1183/09031936.00091407

[B17] Roche N Dalmay F Perez T Kuntz C Vergnenègre A Neukirch F Giordanella J Huchon G FEV1/FVC and FEV1 for the assessment of chronic airflow obstruction in prevalence studies: do prediction equations need revision? Respiratory Medicine 2008 102 11 1568 74 [PMID: 18657959]. 10.1016/j.rmed.2008.06.007 18657959

[B18] Mannino DM Doherty DE Buist AS Global Initiative on Obstructive Lung Disease (GOLD) classification of lung disease and mortality: findings from the Atherosclerosis Risk in Communities (ARIC) study Respiratory Medicine 2006 100 115 22 [PMID: 15893923]. 10.1016/j.rmed.2005.03.035 15893923

[B19] Ekberg-Aronsson M Pehrsson K Nilsson J Nilsson PM Löfdahl C Mortality in GOLD stages of COPD and its dependence on symptoms of chronic bronchitis Respiratory Research 2005 6 98 [PMID: 16120227]. 10.1186/1465-9921-6-98 16120227 PMC1224873

[B20] Celli BR Cote CG Lareau SC Meek PM Predictors of Survival in COPD: more than just the FEV1 Respiratory Medicine 2008 102 Suppl 1 S27 35 [PMID: 18582794]. 10.1016/S0954-6111(08)70005-2 18582794

[B21] Bridevaux P Gerbase MW Probst-Hensch NM Schindler C Gaspoz J Rochat T Long-term decline in lung function, utilisation of care and quality of life in modified GOLD stage 1 COPD Thorax 2008 63 9 768 774 [PMID: 18505800]. 10.1136/thx.2007.093724 18505800

[B22] Calverley PMA Anderson JA Celli B Ferguson GT Jenkins C Jones PW Yates JC Vestbo J investigators TORCH Salmeterol and fluticasone propionate and survival in chronic obstructive pulmonary disease N Engl J Med 2007 356 8 775 789 10.1056/NEJMoa063070 17314337

[B23] Tashkin DP Celli B Senn S Burkhart D Kesten S Men-joge S Decramer M Investigators UPLIFTS A 4-year trial of tiotropium in chronic obstructive pulmonary disease N Engl J Med 2008 359 15 1543 1554 10.1056/NEJMoa0805800 18836213

[B24] Coultas DB Mapel D Gagnon R Lydick E The health impact of undiagnosed airflow obstruction in a national sample of United States adults American Journal of Respiratory and Critical Care Medicine 2001 164 3 372 377 [PMID: 11500335]. 11500335 10.1164/ajrccm.164.3.2004029

[B25] Carter R Holiday DB Nwasuruba C Stocks J Grothues C Tiep B 6-minute walk work for assessment of functional capacity in patients with COPD Chest 2003 123 5 1408 1415 [PMID: 12740255]. 10.1378/chest.123.5.1408 12740255

[B26] Wijkstra PJ TenVergert EM van der Mark TW Postma DS Altena RV Kraan J Koëter GH Relation of lung function, maximal inspiratory pressure, dyspnoea, and quality of life with exercise capacity in patients with chronic obstructive pulmonary disease Thorax 1994 49 5 468 472 [PMID: 8016768]. 10.1136/thx.49.5.468 8016768 PMC474868

[B27] Pinto-Plata VM Celli-Cruz RA Vassaux C Torre-Bouscoulet L Mendes A Rassulo J Celli BR Differences in car-diopulmonary exercise test results by American Thoracic Society/European Respiratory Society-Global Initiative for Chronic Obstructive Lung Disease stage categories and gender Chest 2007 132 4 1204 1211 [PMID: 17934113]. 10.1378/chest.07-0593 17934113

